# Corticospinal and Spinal Excitabilities Are Modulated during Motor Imagery Associated with Somatosensory Electrical Nerve Stimulation

**DOI:** 10.1155/2018/8265427

**Published:** 2018-04-23

**Authors:** E. Traverse, F. Lebon, A. Martin

**Affiliations:** INSERM UMR1093-CAPS, UFR des Sciences du Sport, Université Bourgogne Franche-Comté, 21000 Dijon, France

## Abstract

Motor imagery (MI), the mental simulation of an action, influences the cortical, corticospinal, and spinal levels, despite the lack of somatosensory afferent feedbacks. The aim of this study was to analyze the effect of MI associated with somatosensory stimulation (SS) on the corticospinal and spinal excitabilities. We used transcranial magnetic stimulation and peripheral nerve stimulation to induce motor-evoked potentials (MEP) and H-reflexes, respectively, in soleus and medialis gastrocnemius (MG) muscles of the right leg. Twelve participants performed three tasks: (1) MI of submaximal plantar flexion, (2) SS at 65 Hz on the posterior tibial nerve with an intensity below the motor threshold, and (3) MI + SS. MEP and H-reflex amplitudes were recorded before, during, and after the tasks. Our results confirmed that MI increased corticospinal excitability in a time-specific manner. We found that MI + SS tended to potentiate MEP amplitude of the MG muscle compared to MI alone. We confirmed that SS decreased spinal excitability, and this decrease was partially compensated when combined with MI, especially for the MG muscle. The increase of CSE could be explained by a modulation of the spinal inhibitions induced by SS, depending on the amount of afferent feedbacks.

## 1. Introduction

Motor imagery (MI) is the mental simulation of a movement without muscular activities [1]. MI activates the motor cortical network, such as the primary motor cortex (M1), the premotor cortex, the supplementary motor area, and the parietal cortex [2], a network also involved when the movement is actually executed [3]. Most transcranial magnetic stimulation (TMS) studies have reported an increase of the corticospinal excitability (CSE) during MI in comparison to the rest, as evidenced by an increase of the motor-evoked potential (MEP) amplitude [4, 5]. Recently, the subliminal motor command evoked during MI has been evidenced to reach the spinal level and to modify the excitability of inhibitory interneurons [4].

Most of the previous cited results have been observed using kinesthetic imagery modality, which consists in imagining the actual movement feelings associated with its realization. Indeed, this MI modality has been reported to induce greater CSE increase in comparison to the visual modality [6, 7]. This difference is most likely due to the activation of the somatosensory cortex during kinesthetic MI [8], which interacts with M1 [3]. Interestingly, no somatosensory feedbacks related to the imagined movement are available as no movement is produced during MI. Therefore, the understanding of the interaction between MI and somatosensory feedbacks induced artificially could promote the use of MI for motor performance improvement [9].

Few studies analyzed the interaction between MI and external somatosensory inputs, such as those induced by somatosensory electrical nerve stimulation (SS). Saito et al. [10] measured CSE during an imagined opposition finger task combined with SS (10 Hz, 1 ms pulse width for 20 seconds). They observed an increase of CSE in the thumb muscle when SS intensity was set at the motor threshold. Similarly, Kaneko et al. [11] found an increase of CSE during an imagined index abduction combined with SS (50 Hz, 1 ms pulse width for 2–4 seconds) when the intensity was above the motor threshold in comparison to MI alone. These studies demonstrated the additional influence of SS during MI on upper-limb CSE. However, the SS intensity was set at or above the motor threshold, inducing force development that can modulate MEP amplitude [12]. Indeed, the CSE increase, observed during the stimulation above the motor threshold, could be attributed to the direct activation of the alpha-motoneuron rather than the sole activation of the somatosensory afferent pathway. Indeed, peripheral somatosensory stimulation evoked near the motor threshold activates proprioceptive, sensory and cutaneous afferent fibers [13]. Repetitive activation of these fibers induces inhibitory and/or excitatory neurotransmitter release into the synaptic cleft that can modulate the excitability threshold, that is, the electrophysiological properties of the resting motor neuron membrane. Thus, when a cortical stimulation is induced, the motor neuron may not be in the same state and the MEP amplitude may be affected [14]. This phenomenon is accentuated as the stimulation intensity is high, due to the greater number of fibers activated. In summary, CSE change may be related to the modulation of cortical and/or spinal excitability when the peripheral somatosensory stimulation is near the motor threshold [15]. Therefore, to properly examine the neural impact of the solicitation of afferent fibers during MI, it appears necessary to analyze both corticospinal and spinal excitabilities with SS below the motor threshold that avoids the contamination of the efferent pathway.

In the current study, we conducted a couple of experiments aiming at determining whether the combination of MI and SS below the motor threshold exacerbated the effect of MI on corticospinal and spinal excitabilities. In the first experiment, the participants performed 3 tasks: (1) MI alone, (2) SS alone (65 Hz), and (3) MI combined with SS. We assessed corticospinal and spinal excitabilities at different time points to probe the effects and aftereffects of MI and SS. We hypothesized that MI associated with SS would potentiate to a greater extent corticospinal and spinal excitabilities. We investigated CSE by measuring MEP amplitude evoked by TMS over M1 and spinal excitability by measuring H-reflex amplitude evoked by peripheral electrical nerve stimulation (PNS) over the posterior tibial nerve. In our experimental setup, we applied several PNS with short interstimulus intervals (ranged between 5 s and 10 s) that can affect spinal excitability due to homonymous postactivation depression [16–18]. Therefore, we conducted a second experiment to quantify the effect of successive PNS on spinal excitability at rest and during MI for our specific setup.

## 2. Methods

### 2.1. Subjects

Twelve young healthy adults volunteered to participate in experiment 1 (10 males and 2 females; age 26 ± 8.6 years, height 175 ± 8.7 cm, and weight 72.3 ± 8.8 kg). Data analysis was performed on the data from 11 of the participants, as data from one participant were discarded due to data saving errors. Eight young healthy adults volunteered to participate in experiment 2 (7 males and 1 female; age 28 ± 10 years, height 175.5 ± 3.8 cm, and weight 72.3 ± 7.5 kg), three of them participating in both experiments. Participants had no history of neurological and musculoskeletal disorders. They were normally active and gave their written consent. They did not engage in any strenuous physical activity for at least 24 h before the experimental sessions. All protocols of the current investigation were approved by the University of Burgundy Committee on Human Research and were performed in accordance with the Declaration of Helsinki.

### 2.2. Experimental Setup

#### 2.2.1. Mechanical Recording

Participants sat in a position with hip, knee, and ankle joints placed at a 90° angular position. Measurements were realized on the right calf muscles with the foot secured by two straps to the footplate of a dynamometer (Biodex, Shirley, NY, USA) with the motor axis aligned with the external malleolus of the ankle. Participants were securely stabilized by two crossover shoulder harnesses, and head movements were reduced by a cervical collar strapped to the headrest of the seat.

#### 2.2.2. Electromyographic Recording

The electromyographic activity (EMG) was recorded from two muscles of the right sural triceps (soleus (SOL) and medialis gastrocnemius (MG)) using silver-chloride surface electrodes (8 mm diameter, Ag-AgCl, Mini KR, Contrôle-Graphique S.A., Brie-Comte-Robert, France). Bipolar surface electrodes (interelectrode center-to-center distance of 2 cm) were placed on the midmuscle belly for MG and along the middorsal line of the leg, about 2 cm below the insertion of the gastrocnemius on the Achilles tendon for SOL. The reference electrode was placed between two gastrocnemius muscles of the right leg, below the stimulation site. Before electrode placement, the skin was shaved and cleaned with alcohol to obtain low impedance (<5 kΩ). EMG signals were amplified with a bandwidth frequency ranging from 15 Hz to 1 kHz (gain = 1000) and digitized online at a sampling frequency of 5 kHz using TIDA software (HEKA Elektronik, Lambrecht/Pfalz, Germany).

#### 2.2.3. Transcranial Magnetic Stimulation

A TMS figure-of-eight-shaped conic coil (70 mm loop diameter) was positioned over the left M1 with anteroposterior-directed current orientation to elicit MEPs in SOL and MG muscles of the right leg (Magstim 200, Magstim Company Ltd., Great Britain). To find the optimal site, we stimulated the M1 area of the triceps surae muscle by starting from 1 cm posterior and 1 cm lateral to the vertex of the participant's head and using the lowest stimulation intensity that evoked the greatest amplitude in the SOL and MG muscles. Once the optimal site was found, a mark was placed on the scalp to ensure consistency between stimulations. The coil was then secured by using a homemade tripod with a lockable articulated arm (Otello Factory, T&O brand, France). Then, we realized a recruitment curve at rest to determine the optimal stimulation intensity. The stimulation intensity was increased by steps of 5% of the maximum stimulator output (MSO), and four consecutive stimulations were applied at the same intensity. The optimal intensity was defined when evoking the greatest and the less variable MEP amplitudes on the ascending part of the recruitment curve of both muscles (variation coefficient < 5%). During exp. 1, mean TMS intensity was 72 ± 10% MSO (range: 60 to 98% MSO) corresponding to 131 ± 15% and 124 ± 16% of the rest motor threshold for SOL and MG, respectively. These stimulation intensities are in the range of those classically used in the literature when analyzing the CSE [19].

#### 2.2.4. Peripheral Nerve Stimulation (PNS)

To evoke M and H waves, a single 1 ms rectangular pulse was applied to the posterior tibial nerve using a Digitimer stimulator (model DS7, Hertfordshire, UK). We first placed the cathode electrode stylus in the popliteal fossa and the anode electrode (5 × 10 cm, Medicompex SA, Ecublens, Switzerland) over the patellar tendon, to find the optimal stimulation site, that is, the greatest H-reflex amplitude or M-wave amplitude for the SOL with the lowest stimulation intensity. Once the optimal site was found, we replaced the stylus with a surface electrode (8 mm diameter, Ag-AgCl), secured with a rubber band. Then, we realized a recruitment curve at rest to determine the three optimal stimulation intensities that evoked (1) the lowest EMG response (defined as the rest motor threshold (rMT)), (2) the most reproducible H-reflex, and (3) the maximal M-wave (*M*_max_). For each participant, the stimulation intensity was progressively increased, with a 0.5 mA step, to the *M*_max_ amplitude.

In exp. 1, the mean PNS intensity inducing an H-reflex of about 10–15% of *M*_max_ was 9.1 ± 4.9 mA. The mean PNS intensity was 52.3 ± 20.6 mA corresponding to *M*_max_ wave amplitude of 8.0 ± 4.5 mV. For the somatosensory stimulation (SS), we applied 1 ms monophasic rectangular electrical pulses at 65 Hz for 9 seconds using a second Digitimer stimulator (model DS7, Hertfordshire, UK). The SS intensity was set at 80% of the participants' rMT (mean: 4.9 ± 3.1 mA) to induce afferent inputs without contaminating efferent activation. Due to the electrical noise induced by SS contaminating background EMG, the current was stopped 4 seconds after the beginning of SS for 200 ms to elicit H-reflex or MEP 100 ms after the last SS pulse.

In exp. 2, the PNS intensity to evoke H-reflexes was set at 15% of the *M*_max_ amplitude, to avoid antidromic collisions and to reduce intersubject variability (mean intensity: 10.3 ± 5.1 mA). The PNS intensity to evoke an *M*_max_ wave was set at 54.2 ± 18.1 mA corresponding to an *M*_max_ amplitude of 7.4 ± 4.2 mV.

### 2.3. Experimental Protocol

**Figure 1:**
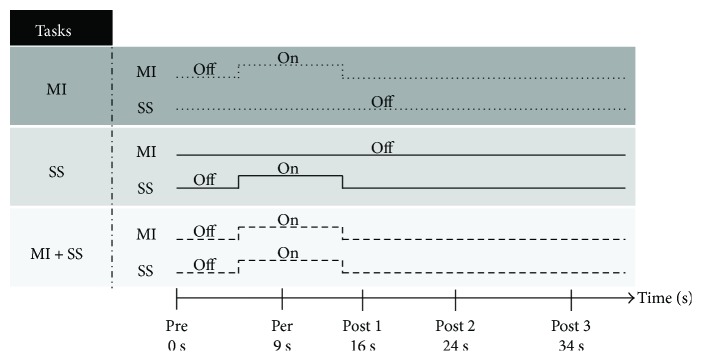
Experimental protocol of experiment 1. Transcranial magnetic stimulation and peripheral nerve stimulation were elicited at several stimulation times: 0, 9, 16, 24, and 34 seconds. Each trial lasted 45 seconds. During the motor imagery (MI) task, participants imagined a plantar-flexion contraction at 50% MVC for 9 s. During the somatosensory stimulation (SS) task, SS was applied for 9 s. During MI + SS, participants imagined the contraction when SS was applied.

The duration of both experiments was about two hours. An overview of exp. 1 is depicted in Figure 1. The first experiment was designed to study the effects of MI associated with SS on corticospinal and spinal excitabilities. To determine maximal plantar flexion force, the participants first performed two maximal voluntary contractions (MVC). If the difference between the two exceeded 5%, an additional trial was performed. The maximal performance was considered for the continuation of the experiment. Then, PNS was applied 4 times at rest to record *M*_max_. To memorize the sensations associated with actual contractions, the participants performed several trials at 50% MVC. A visual feedback helped the participants to match the level of force. Then, we assessed corticospinal and spinal excitabilities during the three tasks: MI only, SS only, and MI associated with SS (MI + SS). All tasks included 8 trials of 45-second duration, half with TMS to elicit MEPs and half with PNS to elicit H-reflexes. The low number of stimulations was chosen to limit the risk of discomfort. A preliminary experiment helped us in determining the number of trials: we found that for 20, 10, or 4 trials, the MEP variation was not significantly different for SOL and MG muscles with 20, 10, and 4 trials (41 ± 27% and 38 ± 16% with 20 trials; 35 ± 21% and 34.0 ± 18% with 10 trials; and 31 ± 28% and 31 ± 23% with 4 trials, resp.). The order of the tasks and of the stimulation type was counterbalanced across participants. TMS and PNS were evoked at 0 s (Pre), 9 s (Per), 16 s, 24 s, and 34 s (Post 1, 2, and 3, resp.). In SS and MI + SS tasks, SS was applied for 9 s (5 s after the first stimulation, i.e., Pre stimulation). In MI and MI + SS tasks, participants imagined a plantar-flexion contraction at 50% of MVC for 9 s. To start and stop imagining, the experimenter gave auditory go (5 s after the Pre stimulation) and stop signals (9 s after the go signal). Therefore, in the MI + SS task, MI and SS were performed at the same time. During MI, participants were instructed to feel the contraction normally generated during actual contractions (kinesthetic modality) and to stay relaxed to avoid muscular contractions.

SS, TMS, and PNS were triggered automatically by the TIDA patch-clamp software (HEKA Elektronik, Lambrecht/Pfalz, Germany) and synchronized with EMG recordings. During the experimental protocol, 60 TMS, 60 PNS, and 16 SS trains were applied.

Exp. 2 was designed to control the effects of successive PNS on H-reflex amplitude at rest and during MI. The experimental setup was similar to exp. 1, without application of SS. As in exp. 1, participants were instructed to stay at rest or to imagine a 50% MVC plantar-flexion contraction. In total, two tasks were performed: (1) PNS induced at 0 s, 9 s, and 16 s during MI (MI Pre-Per-Post) and (2) PNS induced at 0 s, 9 s, and 16 s at rest (Rest Pre-Per-Post). Eight trials were recorded for each task. During the experimental protocol, 48 PNS were applied (24 at rest and 24 during MI).

In both experiments, after each imagined trial, the subjects rated the vividness of their MI using a 7-point Likert scale (from 1 = “very hard to feel” to 7 = “very easy to feel,” 2–6 being intermediate quotes).

### 2.4. Data Analysis

To ensure that the evoked responses were not contaminated by any muscle contraction, the normalized root mean square (RMS) EMG signal was measured 100 ms before each stimulation artefact. When the RMS/*M*_max_ ratio was different from the mean ± 2 SD of the RMS baseline, that is, observed at rest before the first stimulation, the trial was discarded from the general analysis (3% of all trials). Peak-to-peak MEP, *M*_max_, and H-reflex amplitudes were measured during each task for SOL and MG muscles. The ratios MEP/*M*_max_ and H/*M*_max_ were calculated and analyzed.

### 2.5. Statistics Analysis

All data were normalized to *M*_max_ and expressed by their mean ± standard deviation (SD). Data distribution was tested using the Shapiro-Wilk test to ensure the use of the classical analysis of variance for parametric values when appropriate. In exp. 1, all variables were not normally distributed. The Likert scale score during MI and MI + SS was analyzed using a nonparametric Friedman ANOVA.

For both muscles, to ensure that muscles were relaxed, we used four nonparametric related samples Friedman's two-way ANOVAs by ranks with *stimulation* (Pre, Per, and Post 1) and *task* (MI, Rest) 100 ms before each stimulation artefact on EMG RMS/*M*_max_ ratios. We also analyzed MEP/*M*_max_ and H-reflex/*M*_max_ ratios with two nonparametric related samples Friedman's two-way ANOVAs by ranks with *stimulation* (Pre, Per, Post 1, Post 2, and Post 3) and *task* (MI, Rest). When appropriate, we used Wilcoxon's signed-rank tests for paired multiple comparisons applied with a Bonferroni correction.

For exp. 2, all variables were normally distributed for MG but not for SOL. We compared H-reflex ratios using nonparametric related samples Friedman's two-way ANOVAs by ranks for SOL. We used a two-way rmANOVA with *stimulation* (Pre, Per, and Post) and *task* (MI, Rest) for MG. When appropriate, we used Wilcoxon signed-rank tests or paired comparisons Bonferroni's tests, for SOL and MG muscles, respectively. Statistical analysis was performed with SPSS Statistics (2017 version, IBM). The level of significance was set at *p* < 0.05.

## 3. Results

### 3.1. Experiment 1

The vividness of MI, measured with a 7-point Likert scale, was not significantly different between all MI tasks (*χ*^2^ = 6.94, *p* > 0.05). The mean score was 5.3 ± 0.4 and 5.0 ± 0.2 for the MI and MI + SS tasks, respectively. This result ensured that modulations of MEP and H-reflex would not be attributed to the difficulty of task.

#### 3.1.1. EMG Activity

The nonparametric related samples Friedman's two-way ANOVAs by ranks revealed an effect for SOL (*χ*^2^ = 51.98, *p* < 0.01 and *χ*^2^ = 48.12, *p* < 0.01 for TMS and PNS trials, resp.) and MG muscles (*χ*^2^ = 35.88, *p* < 0.01 and *χ*^2^ = 33.27, *p* < 0.01 for TMS and PNS trials, resp.). During SS and MI + SS tasks, SS increased background EMG at Per (for all, *p* < 0.01 compared to Pre and Post stimulations), without an extra increase when imagining (*p* > 0.05). During the MI task, EMG ratios were similar to those at rest (for all, *p* > 0.05). These results ensured that modulations of MEP and H-reflex amplitude would not be attributed to muscle activities (see Table 1).

#### 3.1.2. Corticospinal Excitability

The first stimulation (Pre), induced at the beginning of each trial, was not significantly different between all tasks for both muscles (for all, *p* > 0.05). For MI, SS, and MI + SS, MEP amplitude for the first stimulation was 1.9 ± 1.0%, 2.1 ± 1.0%, and 1.9 ± 0.8% of *M*_max_, respectively, for the SOL muscle and 2.9 ± 1.5%, 3.5 ± 2.0%, and 2.9 ± 1.4% of *M*_max_, respectively, for the MG muscle.

**Figure 2:**
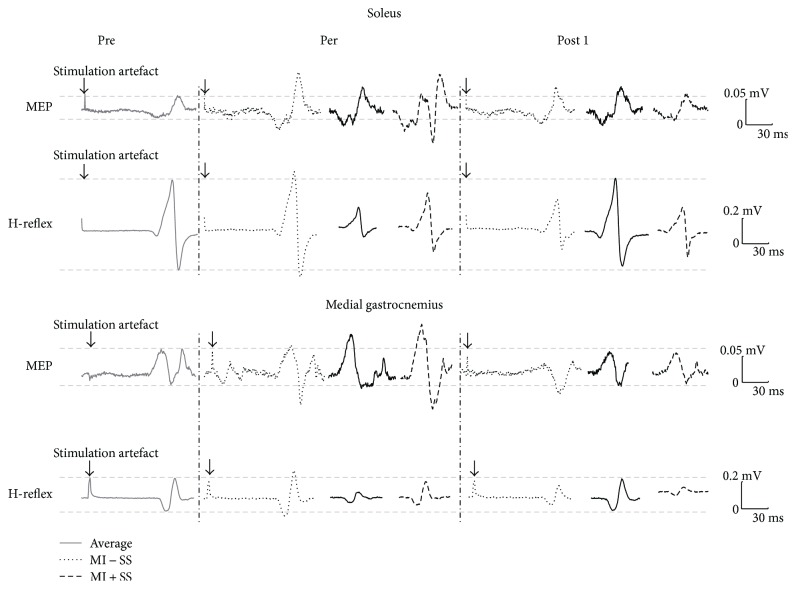
Typical subject. MEP and H-reflex mean responses at Pre, Per, and Post 1 stimulations during MI, SS, and MI + SS for SOL and MG muscles. For both muscles, MEP at Per, when imagining, were significantly greater in comparison to baseline. For MG muscle only, MEP amplitude tended to extra increase during MI + SS in comparison to MI alone. For both muscles, H-reflex at Per decreased during SS but not during the combination with MI.

**Figure 3:**
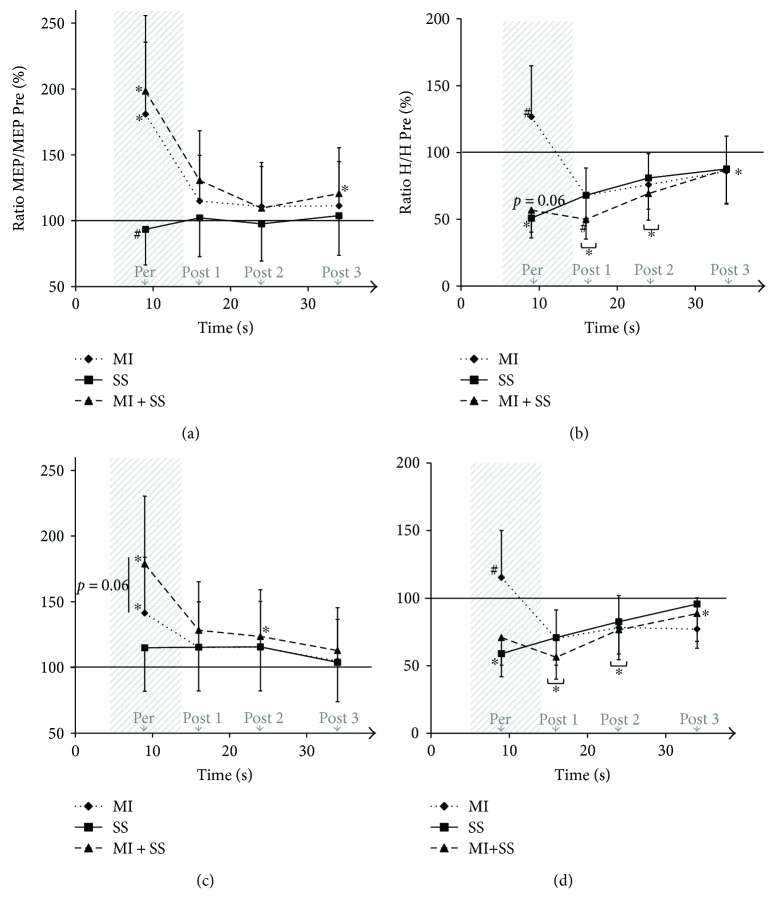
Normalized MEP and H-reflex amplitude. Values recorded in SOL (a and b) and MG (c and d) muscles. MEP amplitude increased when imagining (MI and MI + SS at Per). H-reflex amplitude decreased with SS (SS and MI + SS at Per) and progressively returned to baseline, except for MI + SS. ^∗^Significantly different from Pre test (baseline). ^#^Significantly different from other conditions at the same stimulation time.

For the SOL muscle, a typical trace of one participant was represented in Figure 2. The main results were illustrated in Figure 3(a). The nonparametric related samples Friedman's two-way ANOVA by ranks revealed an effect (*χ*^2^ = 29.72, *p* = 0.008). The Wilcoxon signed-rank tests demonstrated that MEPs increased when imagining with or without SS in comparison to the Pre test value, that is, baseline (at Per: +106 ± 140%, *p* = 0.013 and +81 ± 78%, *p* = 0.026, resp.). After imagining, MEPs returned to baseline from Post 1 for the MI task but not for the MI + SS task. Indeed, MEPs at Post 3 were still above baseline in this task. Note that MEP amplitude was not modulated during the SS task (all, *p* > 0.05).

For the MG muscle, a typical trace of one participant was represented in Figure 2. The main results were illustrated in Figure 3(c). The nonparametric related samples Friedman's two-way ANOVA by ranks revealed an effect (*χ*^2^ = 30.82, *p* = 0.006). The Wilcoxon signed-rank tests demonstrated that MEPs significantly increased when imagining in comparison to baseline (MI task, +41 ± 64%, *p* = 0.041; MI + SS task, +84 ± 66%, *p* = 0.004). Interestingly, MEP increase during MI + SS was marginally greater than that during MI alone (*p* = 0.062). After imagining, MEPs returned to baseline from Post 1 for the MI task (+15%±41%, *p* > 0.05) and at Post 3 for the MI + SS task (+13%±30%, *p* > 0.05). Note that MEP amplitude was not modulated during the SS task (all, *p* > 0.05).

#### 3.1.3. Spinal Excitability

For both muscles, the first stimulation (Pre), applied at the beginning of each trial, did not differ between tasks (for all, *p* > 0.05). For MI, SS, and MI+SS tasks, H-reflex amplitude for the first stimulation was 12.7 ± 12.2%, 14.4 ± 10.0%, and 11.0 ± 6.1% of *M*_max_, respectively, for the SOL muscle and 8.9 ± 8.9%, 8.4 ± 6.1%, and 6.9 ± 5.4% of *M*_max_, respectively, for the MG muscle.

For the SOL muscle, a typical H-reflex trace of one participant was represented in Figure 2. The main results were illustrated in Figure 3(b). The nonparametric related samples Friedman's two-way ANOVA by ranks revealed an effect (*χ*^2^ = 44.93, *p* < 0.001). The Wilcoxon signed-rank tests demonstrated that H-reflex amplitude at Per was significantly depressed in comparison to baseline when SS was applied alone (SS task: −52 ± 54%, *p* = 0.021) and almost depressed when SS was combined with MI (MI + SS task: −47 ± 48%, *p* = 0.062). At Post 1 and 2, H-reflex was still depressed and returned to baseline at Post 3 for the SS task (−12 ± 27%, *p* > 0.05) but not for MI + SS (−13%±20%, *p* = 0.006). Note that H-reflex amplitude was not modulated during MI (*p* > 0.05).

For the MG muscle, a typical H-reflex trace of one participant was represented in Figure 2. The main results were illustrated in Figure 3(d). The nonparametric related samples Friedman's two-way ANOVA by ranks revealed an effect (*χ*^2^ = 32.67, *p* < 0.01). The Wilcoxon signed-rank tests demonstrated that H-reflex amplitude at Per was depressed when SS was applied alone (SS task: −41 ± 42%, *p* = 0.050) but not when SS was combined with MI (MI + SS task: −30 ± 58%, *p* > 0.05). At Post 1 and 2, H-reflex was still depressed, but returned to baseline at Post 3 for SS (−4%±19%, *p* > 0.05) but not for MI + SS (−11%±26%, *p* = 0.016). Note that H-reflex amplitude was not modulated during MI (*p* > 0.05).

For both muscles, the results demonstrated a decrease of H-reflex amplitude at Post 1 in all conditions. For MI + SS and SS alone, this decrease may be due to the stimulation frequency, inducing homosynaptic post activation depression (HPAD) related to the repetitive stimulation of afferent fibers. However, after MI alone, this decrease may be related to a stimulation effect at Per and/or a condition effect. Experiment 2 was designed to examine the influence of successive PNS and condition effects on H-reflex amplitude.

### 3.2. Experiment 2

**Figure 4:**
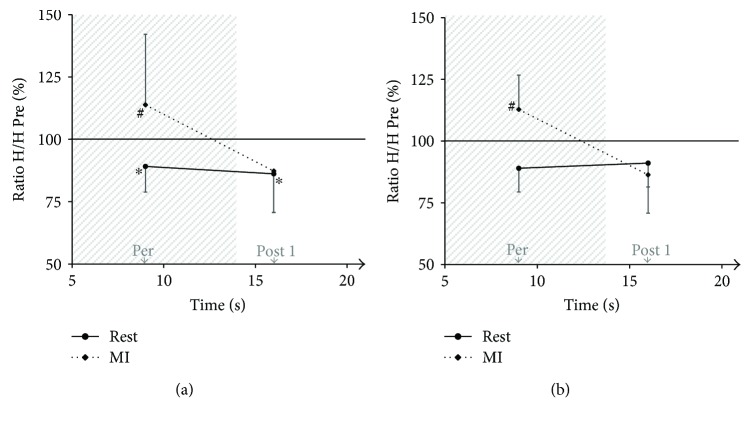
Normalized H-reflex amplitude. Values for SOL (a) and MG (b) muscles. At rest, H-reflex amplitude decreased at Per and Post 1 for the SOL but not for the MG. During the MI task, H-reflex amplitude was not modulated for both muscles. ^∗^Significantly different from Pre test (baseline). ^#^Significantly different from other conditions at the same stimulation time.

The main results of exp. 2 were illustrated in Figures 4(a) and 4(b), for the SOL and MG muscles, respectively.

For the SOL muscle, the nonparametric related samples Friedman's two-way ANOVA by ranks revealed an effect (*χ*^2^ = 11.93, *p* < 0.05). The Wilcoxon signed-rank tests showed that H-reflex amplitudes at Per and at Post were depressed in comparison to baseline when participants were at rest (−11 ± 10%, *p* = 0.036 and −13 ± 18%, *p* = 0.017, resp.). For the MI task, H-reflex at Per, that is, when imagining, almost increased compared to baseline (+13 ± 28%, *p* = 0.069) and was not different from baseline at Post (−14%±19%, *p* > 0.05).

For the MG muscle, the rmANOVA revealed an interaction between *stimulation* and *task* (*F*_1,7_ = 9.24, *p* = 0.003). Bonferroni's post hoc test revealed that H-reflex amplitudes at Per, that is, when imagining, and at Post were not significantly modulated in comparison to baseline (MI task: +12.8 ± 13.9%, *p* > 0.05; −8%±11%, *p* > 0.05, resp.). Note that H-reflex for the rest task was not modulated (*p* > 0.05).

## 4. Discussion

This study was designed to investigate how MI combined with SS modulated corticospinal and spinal excitabilities. The results confirmed that corticospinal excitability increased during MI and MI + SS but not during SS. During MI + SS, MEP amplitude was almost greater than that during MI alone, for MG muscle. On the contrary, spinal excitability was sensitive to SS, during which H-reflex was depressed. However, it was not modulated during MI associated or not with SS. Interestingly, the modulation of corticospinal and spinal excitabilities was muscle dependent.

### 4.1. Corticospinal Excitability

For both muscles, MEP amplitude only increased when participants imagined the plantar-flexion contractions, and not after imagining, which confirms that MI modulates CSE in a temporal-specific manner [20–23].

MEP amplitude during SS was similar to that at rest, showing that the excitatory afferent inputs induced by SS did not affect the corticomotoneuronal transmission efficacy. This result is in accordance with previous studies that applied a short SS duration below the motor threshold [24]. Other experiments using a longer SS duration showed an increase of MEP amplitude [25–28]. Therefore, it appears that the duration of SS seems to play a crucial role to modulate CSE.

MI associated with SS had a tendency to increase CSE to a greater extent in comparison to MI only for MG muscle. This finding may be due to an increase of afferent inputs into M1 at the time of the stimulation. Indeed, through afferent inputs, SS activates the somatosensory cortex (S1), which can mediate the primary motor cortex (M1) activity leading to increased CSE [13, 29]. However, CSE was not modulated by the SS alone, suggesting that SS must be combined with MI to facilitate the interactions between M1 and S1. An alternative explanation would involve the interaction of MI and SS at the spinal level. Grosprêtre et al. [4] recently showed that MI induces a subliminal motor command that modulates the influence of the afferent input to motoneurons at the spinal level, via primary afferent depolarizing interneurons. This interaction likely modulates CSE. The tendency for CSE to increase was not observed for the SOL muscle, suggesting that the difference with the MG muscle could be explained by the amount of afferent inputs recruited during SS at the spinal level.

### 4.2. Spinal Excitability

For both muscles, our results confirmed that spinal excitability was not modulated when imagining [4].

During SS, spinal excitability was depressed. It was demonstrated that the repetition of stimulations decreased the H-reflex amplitude [17] due to a smaller neurotransmitter amount available at the Ia afferent-alpha motoneuron synapse [16]. These inhibitions may originate from the homosynaptic post activation depression effect, the primary afferent depolarization effect, and/or the refractory period of Ia-afferent neurons [30].

When associating MI and SS, the spinal excitability of MG was no longer depressed from baseline. For the SOL muscle, the spinal excitability was less reduced in comparison to SS alone. It seems likely that MI may compensate the inhibitory effects induced by SS. The different behavior between the two muscles could be explained by the amount of afferent inputs recruited during SS. This hypothesis is supported by a lower quantity of neuromuscular spindles in the MG than in the SOL muscle [31, 32] inducing less presynaptic inhibition at the spinal level when SS is applied [33, 34]. Therefore, the subliminal motor command generated during MI that reaches the spinal level [4, 31] may compensate to a greater extent the transmission efficiency between Ia-afferents and motoneurons in the MG muscle. This was observed by a greater reduction of inhibition during MI + SS in this muscle, in comparison to the SOL muscle (Figures 3(b) and 3(d)).

Note that spinal excitability was depressed at posttest right after each task and progressively returned to baseline values. This reduction may be due to successive stimulations elicited to induce H-reflexes and especially to the interstimulation interval, that is, less than 10 seconds inducing presynaptic inhibitions at the spinal level [17]. Indeed, the results of experiment 2 demonstrated that, while participants stayed at rest, the spinal excitability was depressed at Per and Post stimulations in comparison to baseline, with an interstimulus interval of 9 seconds and 7 seconds, respectively. Interestingly, we observed, in experiment 1, a greater decrease of spinal excitability for the MI + SS task at Post 1, that is, right after the task. This greater reduction in the amplitude of the H-reflex after MI + SS may be related to the fact that MI compensates for SS-related inhibitions by reducing presynaptic inhibitions. This mechanism may induce a greater release of neurotransmitters, resulting in a reduction in the amount of neurotransmitters available to respond to Post 1 stimulation versus MI and SS tasks alone.

## 5. Conclusion

The combination of MI and SS exacerbated the effect of MI on corticospinal and spinal excitabilities depending on the afferent inputs elicited by SS at the spinal level. The results of this study were obtained during a single session when the participants were voluntarily engaged in the imagery task with or without SS. We know that MI training or repetitive somatosensory stimulation increases motor performance, specifically muscle strength. It would be of interest to test whether the repetition of the combination of MI and SS facilitates motor performance in comparison to MI and SS alone and to understand the underlying mechanisms.

## Figures and Tables

**Table 1:** Normalized EMG RMS (±SD) in experiment 1. RMS/*M*_max_ ratio is multiplied by 100 and recorded in SOL and MG muscles before (Pre), during (Per), and after (Post 1) motor imagery (MI), somatosensory stimulation (SS), and MI combined with SS (MI + SS) tasks. EMG RMS was measured 100 ms before each stimulation artefact. At Per, during SS and MI + SS (gray boxes), values significantly increased compared to that at Pre and Post 1 (*p* < 0.05).

		SOL			MG		
		Pre	Per	Post 1	Pre	Per	Post 1
MI	TMS	0.79 ± 0.31	0.84 ± 0.37	0.84 ± 0.38	1.6 ± 0.51	1.6 ± 0.47	1.6 ± 0.34
	PNS	0.84 ± 0.38	0.86 ± 0.39	0.84 ± 0.39	1.7 ± 0.56	1.6 ± 0.56	1.7 ± 0.60
SS	TMS	0.75 ± 0.31	5.4 ± 6.2	0.75 ± 0.28	1.6 ± 0.55	13.3 ± 21.9	1.5 ± 0.38
	PNS	0.94 ± 0.69	5.5 ± 6.2	0.95 ± 0.70	1.7 ± 0.56	14.0 ± 25.0	1.7 ± 0.60
MI + SS	TMS	0.79 ± 0.31	5.5 ± 6.2	0.80 ± 0.34	1.6 ± 0.60	13.9 ± 23.8	1.6 ± 0.57
	PNS	0.82 ± 0.33	5.6 ± 6.4	0.82 ± 0.36	1.6 ± 0.56	14.6 ± 26.1	1.6 ± 0.54
